# Inequalities in Global Trade: A Cross-Country Comparison of Trade Network Position, Economic Wealth, Pollution and Mortality

**DOI:** 10.1371/journal.pone.0144453

**Published:** 2015-12-07

**Authors:** Christina Prell, Laixiang Sun, Kuishuang Feng, Tyler W. Myroniuk

**Affiliations:** 1 Sociology Department, University of Maryland, College Park, Maryland, United States of America; 2 Geographical Sciences Department, University of Maryland, College Park, Maryland, United States of America; 3 Population Studies and Training Center, Brown University, Providence, Rhode Island, United States of America; Universidad Veracruzana, MEXICO

## Abstract

In this paper we investigate how structural patterns of international trade give rise to emissions inequalities across countries, and how such inequality in turn impact countries’ mortality rates. We employ Multi-regional Input-Output analysis to distinguish between sulfur-dioxide (SO2) emissions produced within a country’s boarders (production-based emissions) and emissions triggered by consumption in other countries (consumption-based emissions). We use social network analysis to capture countries’ level of integration within the global trade network. We then apply the Prais-Winsten panel estimation technique to a panel data set across 172 countries over 20 years (1990–2010) to estimate the relationships between countries’ level of integration and SO2 emissions, and the impact of trade integration and SO2 emission on mortality rates. Our findings suggest a positive, (log-) linear relationship between a country’s level of integration and both kinds of emissions. In addition, although more integrated countries are mainly responsible for both forms of emissions, our findings indicate that they also tend to experience lower mortality rates. Our approach offers a unique combination of social network analysis with multiregional input-output analysis, which better operationalizes intuitive concepts about global trade and trade structure.

## Introduction

Economic globalization refers to flows of trade and capital among and between countries. Within sociology, a common, critical view on economic globalization is that the historical forces influencing how and when a given country becomes ‘integrated’ into global trade (and the world economy as a whole) conditions the potential paths of development open to that country (e.g.[1]). Here, trade and other relations between countries act as structural mechanisms enabling wealthier, more core countries to maintain favorable terms of trade, which in turn negatively impacts less developed, more peripheral ones in a variety of ways [2,3,4].

In this paper, our primary interest is in tracing how countries’ level of integration in international trade gives rise to between-country inequalities. By integration, we mean the extent to which countries are embedded in the global network of international trade, and we use network analysis to capture this level of embeddedness. A number of scholars (e.g.[5,6,7,8]) use network analysis for measuring countries’ level of integration as an alternative to measures based on exports and/or imports over GDP, noting that such network measures better incorporate implicit notions of ‘economic integration,’ namely, the number and volume of trading ties, as well as the structure of regional trading, that demonstrate the level of connectivity, and hence ‘integration’ in the world economy.

By between-country inequalities, we are interested in emissions, wealth and mortality. For emissions, we make use of multiregional input-output (MRIO) analysis to distinguish between two forms of emissions, these being emissions produced within a country via that country’s manufacturing activities (referred to as *production-based emissions*), and emissions that is triggered by a country’s purchase by accounting for all emissions that is triggered throughout the whole global production chain and then allocated to the final consumer (referred to as *consumption-based emissions*). As means of an example, when a person purchases a toothbrush in the USA, this purchase triggers a supply-chain of production around the entire globe. Some parts hail from Asia, others from Europe, and all get shipped to Northern America for assembly [9]. At each step in this global supply-chain, some form of air polluting emissions occurs, be it through manufacturing, assembly, or transportation processes. In a production based accounting approach, the emissions occurring within a specific country’s border, as it relates to the toothbrush’s production, would be assigned to that country. In contrast, with a consumption-based approach, all emissions along the global commodity chain of the toothbrush would be allocated to the country where the toothbrush was purchased. By accounting for emissions from these two approaches, we are able to distinguish between countries that generate emissions through their production activities, and those that trigger emissions through their consumption. In combination with the use of network analysis, which captures structural features of global trade networks, we see our use of MRIO analysis as providing a more powerful framework that reflects the globalized nature of emissions-intensive commodities.

In addition to better understanding the globalized nature of emissions, we are interested in how emissions and wealth work together to affect countries’ mortality rates. Whereas production-based emissions are often experienced as a ‘burden’ that local populations must endure in exchange for participation in global trade [10], economic growth and wealth are often seen as the main, potential benefits of such participation [11,12,13,14]. These trade-offs come together differently for different countries, such that the potential impacts of emissions experienced by local populations’ may be ‘buffered’ by the benefits associated with more wealth. We consider whether such wealth indeed translates into a buffer against emissions impacts by considering how countries’ share of global production-based emissions stands in relation to their share of global value added. Efficiency is often seen as one sign of a country’s level of development, in that more economically advanced societies have accumulated more wealth to invest in cleaner, more efficient technologies (e.g. [15]) such as better filter technologies, less emissions intensive production structures, and a less polluting fuel mix.

Finally, in looking at how emissions impacts countries’ populations, we have chosen to use a regional pollutant, in this case sulfur dioxide (SO2). This toxic gas is emitted via the combustion of fossil fuels in power plants and manufacturing facilities, and evidence suggests that local exposure to SO2 is linked to respiratory illnesses such as bronchitis or terminal lung cancer in both children and adults [16,17,18,19]. For these reasons, we make use of SO2 (as opposed to a more global pollutant such as carbon dioxide) for our study.

Taken together, our paper makes a number of contributions to the literature on international trade and emissions allocation. Although we are not the first to untangle production-based from consumption-based emissions [20,21,22] a common shortcoming of this work is that it remains largely descriptive, demonstrating the regional and between-country emission disparities without attempting to explain them statistically (although see [23] for an exception to this trend). In addition, we are aware of no other paper that simultaneously attempts to uncover the drivers of these different forms of emissions, as well as trace their effects on countries’ populations.

The rest of the paper is structured as follows: we offer a review on economic and sociological literature on global trade, economic globalization and emissions. This is followed by a description of our longitudinal data, which includes country-by-country trade data on the sector level, as well as a number of country-level attributes. We discuss our methods, which include social network analysis (SNA), multi-regional input-output (MRIO) analysis, and panel data regression models and estimation techniques. We conclude with a discussion and reflection of the study, highlighting our methodological and substantive contributions to ongoing discussions pertaining to the WS and the environment.

## Global Trade, Emissions, and the Effects on Mortality

International trade is often described as a system of increasing interdependent economic relations [6,14]. These economic relations form patterns, giving rise to structural features that shape characteristics and outcomes for countries. A number of studies exist that adopt a network approach to studying international trade relations [5,12,24,25,26]. Some of these studies use network measures to describe the entire network structure, and in doing so, attempt to gauge the extent to which the global economy has become integrated overtime [5,12,27]. Other studies consider how individual countries are conditioned by their position within this global trade network [24,25,26], and/or their level of centrality [28]. Here, an important distinction is made between countries that are positioned in the network ‘core’ versus its ‘periphery.’ In network terminology, a core-periphery structure refers to a two-class partitioning where the core consists of a set of actors (or nodes) that are densely connected to one another and central to the entire network, i.e. they form a well-integrated block and share a similar set of ties to others in the network. In contrast, the periphery refers to a class of countries that are more or less isolated from one another and linked to the rest of the network mainly via ties to the core.

Within this core-periphery structure, countries situated within the core are seen as being more integrated into the overall global trade network, and consequently accruing larger benefits, principally in the form of economic growth and/or development [26]. Here, the well-integrated core is understood to exploit the periphery in an unequal exchange, such that financial investment and/or high-value goods flow from the core to the periphery, in exchange for undervalued goods produced in, or extracted from that region [29,30,31]. Such unequal exchanges, moreover, prompt higher levels of emissions and resource exploitation in these less-developed, more peripheral nations [26,30,32,33,34,35]. In addition, less-integrated, less-developed countries are looking for opportunities for economic growth, which often includes attracting foreign investment and/or the relocation of certain manufacturing activities from core-based transnational companies. As such, these countries’ regimes take a number of measures to attract this economic activity, such as relaxing labor laws and/or environmental regulations, with the consequential result that more environmental degradation is often experienced in these regions [10,35,36,37,38,39].

Another body of research suggests, however, that populations of well-integrated, core countries tend to emit high amounts of emissions [23,28,40]. Here, emissions are seen as rising because of the high presence of energy/pollution-intensive activities found within wealthier, more integrated countries. Although agents within these countries may indeed invest and develop more efficient, less polluting technologies [15], the gains in efficiency are understood to lead to increases in the overall rate of consumption, thus leading to increases in overall resource use and the pollution emissions associated with this [41]. In a similar way, research that adopts a consumption-based approach to pollution suggests that wealthier, more countries are the biggest emitters [20,21,26,42]. Here, wealthier, more integrated countries are seen as consuming a larger global portion of goods and services, and thus, triggering a disproportionately higher amount emissions through their consumption of goods and services. As such, they are seen as being more accountable for a larger share of global emissions.

Taken together, the contrasting narratives presented above offer an unclear picture regarding the extent to which being integrated in global trade conditions the distribution of emissions among countries. Are core countries generating most of the emissions? Or have they managed to decrease levels of production-based emissions through externalization, thus prompting the same (or more) amounts of emissions in other countries? Finally, are the use of cleaner technologies truly capable of combating the negative environmental impacts of high-consuming countries? Part of the confusion in understanding just how much emissions are caused by these more integrated countries may lie in the fact that most studies fail to disentangle, within the context of the same study, pollution that is produced within a countries’ own boundaries (i.e. production-based emissions) and pollution emissions that are triggered through countries’ purchases and consumption habits (i.e. consumption-based emissions). In this paper, we contrast both types of pollution emissions and examine the extent to which being integrated, or ‘core’ conditions these two forms of pollution. In doing so, we seek to offer a better view of the intertwined, interdependent nature of pollution burdens and pollution responsibilities.

In addition to disentangling which countries are the main producers of SO2, and which ones trigger the most SO2 through consumption, we are equally concerned with identifying who is most affected by this pollution, and how a country’s level of integration might help mitigate the impacts of this pollution. As core countries tend to accumulate more wealth than less-integrated ones (e.g. [24,26,26]), the fungible nature of this wealth should help societies adjust and potentially buffer the harmful effects of emissions.

To assess whether such wealth translates into an economic buffer that lowers the negative impacts of emissions, we consider how countries’ share of global production-based emissions (SO2prod) stands in relation to their share of global value added. In doing so, we are essentially looking at countries’ relative efficiency scores, and asking to what extent such efficiency can help mitigate negative impacts of emissions. In assessing the negative impacts of emissions, we consider countries’ mortality rates, in particular, child and infant mortality, as infants and children are generally seen as the most vulnerable segments of a society [43], and hence, the ones most vulnerable to air pollution occurring within non-core countries. Research has shown that infants and children born in low socioeconomic conditions have lower access to resources and health services, and at the same time higher exposure to pollutants, thus increasing their risk for preterm births and premature death [44]. In addition, research on water pollution and infant mortality [45] and children’s health [46] has shown that the periphery suffers higher mortality rates than the core, and such high mortality rates are generally seen as resulting from the multiple structural disadvantages found within the periphery, namely weaker institutions and less environmental safe-guards [35,46,47,48].

Taken together, we predict that more efficient countries would be ones that would also hold lower mortality rates, regardless of how much that country might actually pollute. Stated differently, we argue that countries with higher shares of wealth (in relation to shares of emissions) have an economic buffer that would help mitigate the harmful impacts of pollution on infant and child mortality.

## Material and Methods

This section describes our dataset, our variables and measures, the methods for constructing these measures, and panel regression techniques.

### Data and Data Transformations

Our trade data were extracted from the Eora database. Eora is a multi-region input-output database that provides a time series of high resolution input-output (IO) tables with matching environmental and social satellite accounts for 186 countries [49]. The MRIO tables from Eora contains trade flows, production, consumption and intermediate use of commodities and services for 26 sectors, both within and between 186 countries (see http://www.worldmrio.com/ for more details). Our data covers a 20 year time span (1990–2010).

The benefit of using the global MRIO data, (combined with the SO2 data described below), is that we are able to calculate how SO2 is triggered by both consumption and production processes along the entire global production chain. As part of our intention here is to distinguish between consumption-based and production-based emissions on the global level, such detailed economic trade data are necessary.

Although Eora contains data on 186 countries and regions, we were not able to find corresponding data for our other variables of interest for all countries in this dataset, as some countries in Eora have been aggregated into supranational regions. In spite of this constraint, we managed to gather health data for 172 of the 186 cases (see the full listing in the SI).

For calculating value-added (VA) and consumption-based SO2, we made use of the full multi-regional input-output (MRIO) database without any transformations. For calculating countries’ level of integration, however, we transformed the dataset, following guidelines established by previous sociological work on global trade networks [24,50]: first, we aggregated all 26 sectors to form one country-by-country trade matrix consisting of valued data. A single, valued trade network dataset is often preferred for operationalizing ideas of coreness or integration, as a country’s position is determined not only through the quantity and patterning of trading ties to others, but also considers the trade volume of those ties [28]. In addition, as rows in the trade matrix correspond to exports and columns to imports, we took the trade matrix and its transpose, then summed the two together to arrive at a symmetrized matrix that combines, for each country, information on that country’s exports and imports. By summing the export matrix to its transpose (i.e. the import matrix), we focused attention on the structure of trade, as opposed to the directionality of trade ties (18, 22). Lastly, to smooth out any skewness, we took the square root of each cell in the symmetrized matrix.

Our SO2 emission data are at the sector level, and were also collected from the Eora database.

One thing to note about these SO2 data is that they refer to the emissions themselves, and not to concentrations of SO2 emissions. Concentrations of SO2 take into account whether or not air filters and other cleaning technologies are used to trap the emissions and lessen the amounts that escape into the atmosphere. Analyses of SO2 and economic or health implications are often limited to examining SO2 emissions [51,52] rather than their concentrations. Concentrations would be better correlated to health impacts but less so to production or consumption based emissions due to other ambient and atmospheric factors and cross-boundary pollution. As SO2 concentrations data are not available for many countries in our sample, we are left with examining SO2 emissions.

### Measures

A number of analysts have used social network analysis (SNA), a methodological approach for analyzing relational data [53], to measure structural ideas of global trade (e.g. [24,54,55,56,57]). Although no single network measure has been widely adopted for assessing countries’ level of integration in global trade (see [58] for review), our use of SNA is in keeping with recent research focusing on global trade networks (e.g. [24,26,50]). In particular, we made use of the continuous coreness procedure [59] for measuring countries’ level of integration within the world trade network. This procedure fits a core/periphery model to an observed network to identify the extent to which the observed network approaches an ideal core/periphery structure. In an ideal core/periphery structure, the ‘core’ is a block of actors who are tied to one another, and in addition, have ties with many other actors in the network. To be core, then, is to be highly central in one’s own right, as well as part of a dense block of other highly central actors. In contrast, peripheral actors form a second block in which members are largely isolated from one another, and any ties they do hold are with the core. The coreness procedure introduced by Borgatti and Everett [59] proceeds in determining what type of partitioning of actors (in our case, countries) in the observed network most closely brings that network towards an ideal core/periphery partitioning. The procedure results in a vector of scores assigned to countries, which range between 0 and 1, the higher values indicating a country being more core, and lower values indicating the country being more peripheral. The advantage of this procedure is that it results in a ratio-scaled vector of scores, thus enabling a higher degree of precision in making cross-country comparisons. We refer to this resulting vector of scores as our level of ‘integration’ measure for countries. A full listing of all countries and their integration/coreness scores can be found in S1 Dataset.

Our production-based SO2 measure consists of the data collected from the Eora database. Our consumption-based SO2 measure was constructed using MRIO Analysis (see [60] for an additional example). At its core, MRIO analysis is an accounting procedure relying on national economic input-output (I-O) tables (the global MRIO dataset consists of 186 such national I-O tables) and international trade matrices, depicting the flows of money to and from the various sectors of the national and international economies, thus revealing each sector’s entire supply chain. This method has been applied to global trade studies on land use [61], water consumption [62,63], CO_2_ emissions [64,65], materials use [66], and biodiversity loss [67]. The process begins with the Leontief inverse of the MRIO matrix, which essentially involves multiplying an economy’s input requirements with each other by an infinite number of times, thus representing infinite rounds of production layers triggered by the previous set of inputs to fulfill production for final consumption. As such, computing the matrix inverse accounts for all the direct and indirect inputs triggered by final demand for a consumption item in any given country. Second, we calculated the total input requirements to satisfy final demand by multiplying the inverse matrix by final demand of a particular consumption item in any country. Finally, to account for total consumption-based SO2 emissions, direct pollution coefficients for each sector and each economy, showing the amount of pollution that is created for the production of each unit of economic output, are multiplied with the total inputs that are triggered by final demand. Technical details on consumption-based emissions can be found in S1 Supporting Information.

For measuring mortality, we used child and infant mortality data (each per 1000 live births) downloaded from UNICEF’s Millennium Development Goals database (please visit http://mdgs.un.org/unsd/mdg/Data.aspx for details). We included these measures to capture the potential impacts of SO2 and a country’s level of integration on vulnerable segments of a country’s population, i.e. children under the age of 5 and 1. In our regressions, we used the natural logarithm of these two mortality variables in order to normalize their distributions.

To measure countries’ efficiency levels, we divided countries’ share of global SO_2_ emissions (%SO_2_) by their global share of value-added (%VA), such that (NEM = %SO_2_/%VA). As such, this NEM essentially translates as a *normalized* measure of a country’s efficiency, as all countries are being assessed in relation to global totals. This enables us to see how countries compare in terms of *global* shares of emissions and wealth, as opposed to comparisons based on individual country attributes, as is found in more traditional efficiency measures (e.g. SO_2_/GDP).

Our major control variables include population size (in 1,000,000s) and urbanization (percentage of population estimated to live in urban areas in a country), both taken from the World Bank’s database (http://www.worldbank.org). Research has shown that population size is positively linked to forms of environmental degradation, including air pollution [68,69]. Similarly, research has shown a positive link between a country’s level of urbanization and environmental degradation [29,69,70]. Urbanization has also been shown to be positively linked to infant mortality in the periphery [46], although in the core, urban centers have historically been places where wealth and other key resources necessary for a higher quality of life become concentrated (e.g.[71]), thus potentially mitigating the health threats of air pollution. In addition, we have also included countries’ health expenditure as percentage of GDP and countries’ fertility rates as control variables for regression models predicting infant and child mortality. Data for both these variables was downloaded from the World Bank (http://www.worldbank.org).

All variables’ means and standard deviations are shown below in Table 1.

**Table 1:** Descriptive Statistics for all Variables.

Variable	Mean and Standard Deviation (SD)	Observations
Consumption-based SO_2_ (ln)	4.65 (2.01)	3591
Production-based SO_2_ (ln)	4.6 (2.2)	3486
Integration (ln)	-2.6 (1.8)	3591
Pollution-Wealth Ratio (ln)	0.49 (1.4)	3483
Population Size (ln)	1.9 (1.9)	3609
Urbanization	54.04 (23.51)	3612
Under 1 Mortality (ln)	3.14 (1.08)	3612
Under 5 Mortality (ln)	3.41 (1.17)	3612
Fertility rates (ln)	3.23 (1.69)	3483
Health expenditure as % of GDP	3.49 (1.96)	2710

Data gathered for years 1990–2010 for 172 countries. The number of observations differ across the variables, as some variables did not have data for certain years. Variables were logged to handle skewness.

### Regression Analyses

For assessing which countries are causing SO_2_ pollution, we regressed countries’ integration scores against our two SO_2_ pollution measures, controlling for population size and urbanization. For assessing the link between countries’ efficiency (NEM), and their level of integration, we regressed countries’ integration scores on their NEM, with the same controls. Finally, to gauge the extent to which countries suffer from their pollution costs, we regressed mortality rates on levels of integration, NEM, and production-based SO_2_, alongside other controls.

Our actual regression technique was a time-series cross-sectional Prais-Winsten estimation technique with panel-corrected standard errors (PCSE) as used by Jorgensen and Clark (2012). This technique allows for disturbances that are heteroskedastic and contemporaneously correlated across panels. The PCSE correction is capable of avoiding extreme overconfidence often associated with the popularly-used feasible generalized least-square estimator in the case of panel data sets in which the total time period *T* is smaller than total sections *N*. We control for common first-order autocorrelations of the disturbance terms and both period-specific and unit-specific disturbances. Because there are missing values in different places across variables, we employed ‘pairwise’ option associated with the xtpcse command in Stata (ver. 13) to include all available observations with non-missing pairs, thus maximizing the number of observations in the unbalanced panels.

**Fig 1:**
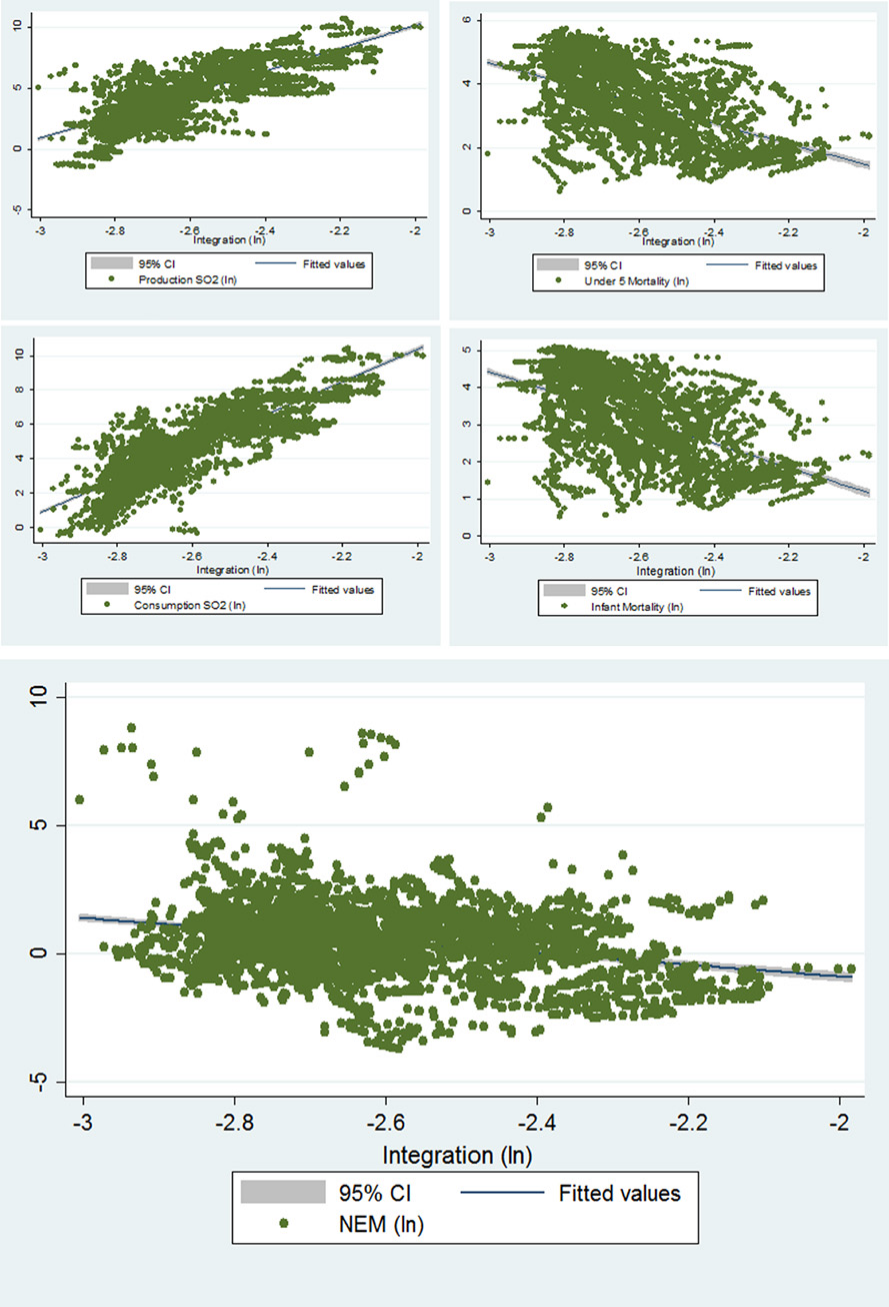
Integration Plotted against Pollution and Mortality Outcomes. Starting from the upper left-hand corner, and moving clockwise, the scatterplots shown in Fig 1 demonstrate the linear relationships between countries’ logged integration values (found on the x- axis of each plot) and the logged values (found on the y-axis) for i) Production-based SO2, ii) Under 5 Mortality, iii) Infant Mortality, iv) Normalized Efficiency Measure, and v) Consumption-based SO2.

Prior to running regression models, we plotted our main predictor (countries’ level of integration) against our main outcome variables. These scatterplots are found below in Fig 1.

## Results

**Fig 2:**
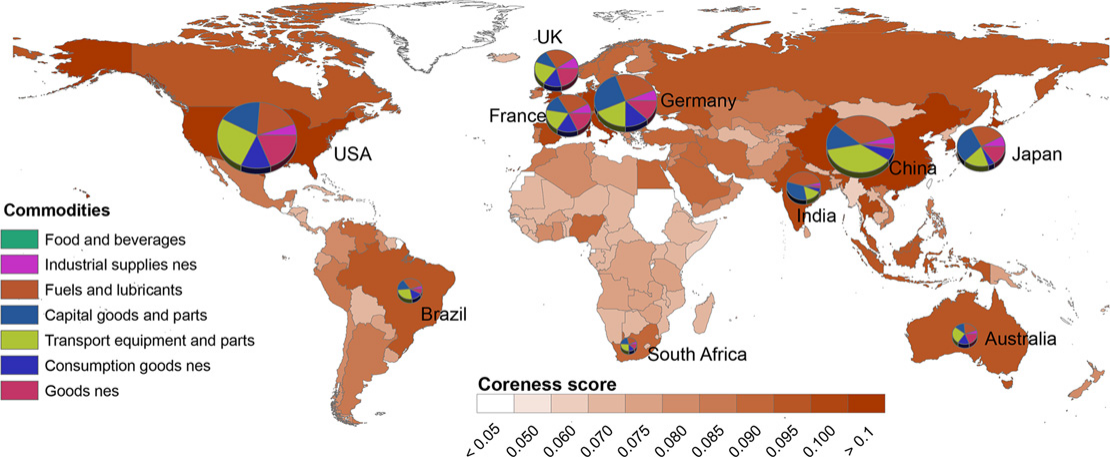
Global Map showing Countries’ Integration and Patterns of Imports. Taken together, the map gives a heuristic sense for some of the global trade patterns, showing how well-integrated countries consist of both developed (e.g. USA, Germany and Japan) and developing (e.g. China and India) economies, and how these well-integrated countries differ according to their import patterns.

To begin, we offer a global map (Fig 2) showing countries’ level of integration according to year 2010, where darker shades indicate higher levels of integration. The pie charts depict well-integrated countries’ total imports, again for year 2010.

Next, we present our regression results, starting with results for production-based SO_2_ and consumption-based SO_2_, as shown below in Table 2.

**Table 2:** Pollution Regressed on Countries’ Level of Integration.

	Production-based SO_2_ (ln)		Consumption-based SO_2_ (ln)	
	Model 1a	Model 2a	Model 3a	Model 4a
	*b*	*b*	*b*	*b*
Integration (ln)	1.067*	1.175*	2.673*	3.409*
	(0.249)	(0.215)	(0.192)	(0.215)
Population (ln)		0.904*		0.670*
		(.015)		(0.023)
Urbanization		0.032*		0.022*
		(0.001)		(0.001)
Constant	6.285*	4.428*	11.035*	11.305*
	(0 .690)	(0.616)	(0.531)	(0.615)
Fixed?	Year, country	Year^#^	Year, country	Year^#^
Observations	3,486	3,483	3,591	3,588
Wald χ^2^	36837.92	87103.48	11710.32	238155.10
R-squared	0.965	0.815	0.97	0.866

* p < 0.01. These are unstandardized *b* values. Standard errors in parentheses.

^#^ The introduction of country fixed effect led to a highly singular variance matrix, implying high collinearity.

Here, we see that, for both emission outcome measures, ‘integration’ holds a positive, highly significant coefficient, even after the controls are entered into the model. This finding suggests that the level of countries’ SO_2_ pollution is positively associated with their level of integration in global trade. In addition, consumption-based SO_2_ is more strongly correlated to integration than production-based SO_2_. In particular, models 2a and 4a show that the integration coefficient (elasticity) is much stronger (i.e. more than doubled) in relation to consumption-based SO_2_ than production-based SO_2_, once we control for population size and urbanization. Finally, the explanatory power of the models increases once the control variables are introduced, as reflected in the R^2^ values. As such, we see evidence that core countries are responsible for larger pollution emission levels, relative to less core countries, both through their at-home manufacturing activities, and also, through their consumption habits. Our findings thus suggest support for both our narratives: core, well-integrated countries are both the larger emitters of SO_2_ emissions via manufacturing, and they also appear to be the main ‘externalizers’ of emissions, in this case via consumption.

To assess whether being more integrated translates into attaining a higher ‘buffer’, we turn to results presented in Table 3. Here, we note the significant, positive relationship between levels of integration and NEM scores, both with and without controls.

**Table 3:** Countries’ Normalized Efficiency Level Regressed on their Level of Integration.

	Model 1	Model 2
Integration (ln)	.9481**	.532**
	(.3466)	(.297)
Population (ln)		1.121***
		(.160)
Urbanization		.036***
		(.003)
Constant	3.166***	-1.794
	(.959)	(1.00)
Fixed?	Year, country	Year, country
Observations	3,483	3,480
Wald χ^2^	18439.45	354704.56
*R* ^2^	0.822	0.833

** p < 0.05

*** p < 0.01.

These are unstandardized coefficients. Standard errors in parentheses.

These findings indicate that as countries become more core, they gain larger shares in global SO_2_ than shares of wealth (regardless of how much they might actually emit). Thus, there appears to be a tendency for more integration leading to less efficiency, i.e. *not* developing an economic buffer against emissions.

To what extent does an increase in efficiency have a pay-off, in terms of mortality rates, for individual countries? Integration does not seem to go hand in hand with a lower NEM score, but would lower NEM scores translate into an economic buffer to help reduce mortality rates for infants and children? Table 4 below shows regression model results exploring this question.

**Table 4:** Integration Predicting Infant and Child Mortality.

	Under Age 5 Mortality		Infant Mortality	
	per 1000(ln)		per 1000 (ln)	
	Model 1	Model 2	Model 4	Model 5
Integration (ln)	-.0003	-.394*	.001	-.5481**
	(.024)	(.131)	(.026)	(0 .155)
NEM (ln)		.0501**		.072**
		(.012)		(.0116)
Product- SO_2_ (ln)		.017		.011
		(.012)		(.013)
Fertility (ln)		1.242**		1.117**
		(.054)		(.051)
Health % GDP		-.034**		-.044**
		(.006)		(0.007)
Urbanization		-.016**		-.014**
		(.001)		(.001)
Constant	5.291**	2.058**	4.912**	1.452*
	(.071)	(.401)	(.074)	(0 .474)
Fixed?	Year, country	Year^#^	Year, country	Year^#^
N or Observations	3,591	2,567	3,591	2,567
Wald χ^2^	721148.80	23563.60	640638.06	24370.93
R^2^	0.989	0.925	0.9895	0.936

* p < 0.05

** p < 0.01.

These are unstandardized coefficients. Standard errors in parentheses.

^#^ The introduction of country fixed effect led to a highly singular variance matrix, implying high collinearity.

For both sets of models in Table 4, we see that countries’ level of integration holds a negative and statistically significant relationship with mortality rates, suggesting that more core countries have lower mortality rates. In addition, countries’ NEM scores have a positive and statistically significant relationship to mortality, suggesting that countries with higher shares of emissions in relation to shares of VA suffer higher rates of mortality. In short, it appears that more core countries with higher economic buffers (in the form of lower NEMs) experience lower mortality rates. Interestingly, levels of production-based SO2 is not in any way linked to mortality, implying that countries might potentially have high levels of SO2 emissions within their own boundaries, but this is insignificant if the same countries are well-integrated, and have a low NEM score. As such, more core countries may have managed to ‘cloak’ the potential negative impacts of SO_2_ emissions through making a number of adjustments (a lower NEM score, more urbanization, more spending on health, and so on), and these adjustments act as causal mechanisms to buffer the real impacts of SO_2_ on the population.

We also note that our controls operate largely as expected: i) the urbanization coefficient is negative and significant, suggesting that countries with higher portions of an urban population experience lower mortality rates; ii) the health expenditure coefficient is also negative and statistically significant, implying that higher spending on health per unit of GDP coincides with fewer deaths, and iii) fertility rates are positively and significantly linked to mortality, implying that countries with more births tend to suffer more deaths.

Taken together, when we ask ourselves who is most affected by SO2 emissions, our findings offer a complex picture. More integrated countries experience lower mortality rates, yet they appear to do so via a number of mechanisms. One such mechanism is a country’s NEM score. Low NEM scores (implying greater efficiency) appear to help reduce mortality, and thus, it appears that higher levels of integration help countries acquire a stronger economic buffer to mitigate the negative impacts of SO_2_ on mortality. In addition, higher levels of integration coincide with higher levels of urbanization, higher health expenditures and fertility rates. All these mechanisms thus appear to be working together to give more core, integrated countries an advantage over less-core ones with regards to mortality rates. Thus, the simple answer to the question, ‘who is most affected by pollution emissions?’ is that less core countries are the ones most affected. Yet it is via a number of causal mechanisms associated with one’s position in the global trade network (as implied by Tables 3–4) that this disadvantage arises.

As robustness tests to these findings, we re-ran the same models shown in Tables 2–4 as stepwise regression models (see Tables 5–7 below).

**Table 5:** Stepwise Regression Results for Two Forms of Pollution.

***Production-based SO2(ln)***				
Variables	Coefficient	Std.Err.	t	p
Integration (ln)	3.185	0.149	21.350	0.000
Population (ln)	0.779	0.013	58.710	0.000
Urbanization	0.024	0.001	25.900	0.000
Constant	9.999	0.440	22.750	0.000
Observations	3,483			
Adjusted R^2^	0.79			
***Consumption-based SO2(ln)***				
Variables	Coefficient	Std.Err.	t	p
Integration (ln)	5.044	0.119	42.440	0.000
Population (ln)	0.566	0.010	54.870	0.000
Urbanization	0.016	0.001	21.440	0.000
Constant	15.864	0.350	45.330	0.000
Observations	3,588			
Adjusted R^2^	0.84			

**Table 6:** Stepwise Regression Results for Countries’ NEM.

Variables	Coefficient	Std.Err.	t	p
Integration (ln)	-5.100	0.183	-27.880	0.000
Population (ln)	0.411	0.016	25.290	0.000
Urbanization	0.006	0.001	5.680	0.000
Constant	-13.952	0.539	-25.880	0.000
Observations	3,480			
Adjusted R^2^	0.23			

**Table 7:** Stepwise Regression Results for Infant and Child Mortality.

***Under Age 5 Mortality per 1000 (ln)***				
Variables	Coefficient	Std.Err.	t	p
Integration (ln)	1.262	0.030	42.130	0.000
NEM (ln)	-0.014	0.001	-22.350	0.000
Product- SO_2_ (ln)	-0.100	0.007	-13.470	0.000
Fertility (ln)	0.045	0.013	3.380	0.001
Health % GDP	-0.789	0.170	-4.640	0.000
Urbanization	0.052	0.013	4.000	0.000
Constant	0.884	0.493	1.790	0.073
Observations	2,567			
Adjusted R^2^	0.77			
***Infant Mortality per 1000 (ln)***				
Variables	Coefficient	Std.Err.	t	p
Integration (ln)	1.091	0.023	46.780	0.000
NEM (ln)	-0.119	0.006	-20.620	0.000
Product- SO_2_ (ln)	-0.012	0.000	-24.050	0.000
Fertility (ln)	0.065	0.010	6.180	0.000
Health % GDP	-1.122	0.133	-8.470	0.000
Urbanization	0.056	0.010	5.550	0.000
Constant	-0.163	0.384	-0.430	0.671
Observations	2,567			
Adjusted R^2^	0.83			

The stepwise regression model results largely replicate our findings presented in Tables 2–4. The main differences between the results/models include the fact that the ‘pairwise’ regression option (Tables 2–4) includes all available observations with non-missing pairs, thus maximizing the number of observations in the unbalanced panels. In contrast, the standard stepwise regression models (Tables 5–7) exclude those observations with missing values. In addition, the t-test in stepwise regression is less efficient, making the results slightly less reliable than those from the pairwise option. As such, although production-based SO2 appears as a significant predictor of mortality outcomes in Table 7, we return to our more conservative result(s) found in Table 4, which show production-based SO2 being an insignificant predictor of mortality outcomes. Other than these noted differences, the patterns in the data results are largely the same.

## Discussion and Conclusion

We began our paper with a distinction between two ways of accounting for SO_2_ emissions: production-based and consumption-based SO_2_. We showed that both ways of accounting for emissions hold a positive, linear relationship with countries’ level of integration. Well integrated countries not only emit larger quantities of SO_2_ than less integrated ones, they also are the ones who trigger the most SO_2_ throughout global supply chains through their consumption habits.

Our second main research aim was assessing, ‘who is most affected by emissions?’ Answering this question involved two steps. First, we looked at the relationship between integration and NEM scores. The literature suggests that being core gives countries a number of ‘spill over’ benefits, and thus, we argued, it is not so much that more integrated countries stop polluting, but rather, that their shares of wealth should exceed their shares of emissions, and such an excess in wealth should be perceived as an economic buffer that outweighs pollution costs. Our results showed that, contrary to what we expected, higher integration levels corresponded with higher NEM scores.

Although more integrated countries tended to have higher NEM scores, our next round of analyses indicated to us that more core, integrated countries with stronger economic buffers appeared to be in a better position to mitigate the negative impacts of pollution, the so-called ‘costs’ of SO_2_ emissions. In particular, an increased economic buffer (in the form of a low NEM) coincided with more urbanization and higher spending on health, all of which could help mitigate the negative impacts of emissions in the form of lower mortality rates. Thus, our findings suggest that being well-integrated works in conjunction with a number of mechanisms–these being countries’ NEM scores, their level of urbanization and amount of health expenditure–to effectively lower mortality rates. In other words, countries’ position within the larger trade network implies a number of spill-over benefits, as suggested by the literature (e.g. [72]).

As a robustness check to our analyses, we re-ran the same set of regression models for predicting air pollution and mortality outcomes, replacing level of integration with two alternatives. These two alternatives included i) a categorical variable for trade network integration and ii) GDP per capita. The categorical measure was the same used in other trade network studies (e.g. [7,55]), and GDP per capita was used to replicate other globalization studies focused on the relationship between emissions and wealth (e.g. [69,73,74]). The details of these variables, their measurements, and the regression results can be found in our SI (see Tables A-D in S1 Supporting Information File). In sum, the results for these alternative models largely replicated those presented here in Tables 2–4, and the R^2^ values were very similar. The main difference between results presented in Tables 2–4 and those based on the two alternative measures is that the coefficients for the alternative measures proved weaker than those using integration.

In terms of our contribution to the literature: although we are not the first scholars to disentangle consumption-based from production-based emissions (e.g. [75,76,77]), we are unaware of any research that has used such an approach to make theoretical arguments about emissions inequalities and mortality outcomes resulting from trade network position in the way we have demonstrated here. By doing so, we have shown, quite clearly, evidence for well-integrated, core countries being both major polluters as well as a major externalizers of emissions. Further, our focus on SO2 has enabled us to see how global trade patterns can have real local impacts in the form of mortality rates. As such, we have moved beyond fundamental concerns pertaining to inequalities resulting from globalization (e.g. wealth and emissions) to show how such inequalities translate into real ‘life or death’ issues for given societies.

Our study has, through a series of analytical steps, slowly developed a picture of the role core countries have on both emissions and mortality outcomes. As data becomes available, future research will track these processes over a broader range of environmental outcomes, such as land displacement, water usage, and other forms of emissions. When thinking about environmental justice, such comparisons between consumption-based forms of environmental degradation and degradation occurring within a country’s boarders are needed for deepening our understanding of who is responsible for and who suffers from environmental harm within the global trade system.

## Supporting Information

S1 Supporting Information (DOCX)

S1 Dataset (XLSX)
