# Supplementary material for: Inequalities in Global Trade: A Cross-Country Comparison of Trade Network Position, Economic Wealth, Pollution and Mortality
Source: PLoS One. 2015 Dec 7;10(12):e0144453. doi: 10.1371/journal.pone.0144453 (PMC4671716; doi:10.1371/journal.pone.0144453)
Supplement: S1 Supporting Information — (DOCX) [file pone.0144453.s001.docx]

**S1 Supporting Information**

The Supporting Information (SI) contains math formulas used in the MRIO analysis and a section on robustness tests.

In a MRIO framework, different countries are connected through global trade. The MRIO framework (shown in Equation 1 below) begins with a technical coefficient matrix *A**, which is calculated by the following equation:

[1] A* = $a_{ij}^{pq}=z_{ij}^{pq}/x_{j}^{q}$

where inter-sector monetary flows, z, occur from sector i in country p to sector j in country q;

$x_{j}^{q}$ refers to the total output of sector j in country q;, Final demand matrix *Y** consists of $y^{pq}$, where $y^{pq}$ refers to each sector’s output (y) produced in country p directly consumed by the final user in a given country. Finally, x* is a vector of sectoral outputs in all countries. The technical coefficient matrix A*, final demand Y* and sectoral outputs x* can be displayed as follows:

$A^{*}=\left[ \begin{matrix} A^{11} & A^{12} & \begin{matrix} \cdots& A^{1n} \end{matrix} \\ A^{21} & A^{22} & \begin{matrix} \cdots& A^{2n} \end{matrix} \\ \begin{matrix} \vdots\\ A^{n1} \end{matrix} & \begin{matrix} \vdots\\ A^{n2} \end{matrix} & \begin{matrix} \begin{matrix} \ddots& \vdots\end{matrix} \\ \begin{matrix} \cdots& A^{nn} \end{matrix} \end{matrix} \end{matrix} \right]$; $Y^{*}=\left[ \begin{matrix} \begin{matrix} y^{11} & y^{12} & \cdots\\ y^{21} & y^{22} & \cdots\\ \vdots& \vdots& \ddots\end{matrix} & \begin{matrix} y^{1n} \\ y^{2n} \\ \vdots\end{matrix} \\ \begin{matrix} y^{n1} & y^{n2} & \cdots\end{matrix} & y^{nn} \end{matrix} \right]$ ; $x^{*}=\left[ \begin{matrix} x^{1} \\ x^{2} \\ \begin{matrix} \vdots\\ x^{n} \end{matrix} \end{matrix} \right]$;

Therefore, the MRIO framework can be written as:

[2] $x^{*}=A^{*}x^{*}+y^{*}$

To solve for *x*, we obtain

[3] $x^{*}={{(I-A}^{*})}^{-1}y^{*}$

where *(I-A*)^-1^* is referred to as the Leontief inverse matrix, which captures both direct and indirect inputs to satisfy one unit of final demand in monetary values; *I* is the identity matrix with ones on the diagonal and zeros on the off-diagonal.

To calculate the consumption based pollution, we extended the MRIO table with SO2 emissions coefficients, as shown in equation 4.

[4] $G=e^{*}{(I-A^{*})}^{-1}Y^{*}+hh$

Where G is the total SO_2_ emissions; and *e** is a vector of direct SO2 emissions per unit of economic output; *hh* is the household direct emissions.

**Robustness Tests**

This section contains detailed information on the robustness tests we conducted for results found in the main body text of our article, Tables 2-4. These consist of the following: one set of tests in which we replaced the ‘integration’ measure found in our main article with two alternatives: GDP per capita and a categorical measure of network position. The second set of tests are stepwise regression models that largely replicate the findings found in Tables 2-4 of the main article.

Beginning with the first set, models using GDP per capita are found in Tables S1-S2 and results for the second, the categorical measure of network position, are found in Tables S3-S4. The data for GDP per capita was downloaded from the United Nations Statistics Division (<http://unstats.un.org/unsd/default.htm>), and are in constant 2005 prices for US dollars.

The procedures for attaining our categorical network position guidelines established by Mahutga (2006) and Mahutga and Smith (2011) in their analysis of international trade data. In particular, we used a positional analysis procedure to assign countries a network position. A positional analysis procedure estimates the extent to which actors (in our case countries) in a network share similar patterns of relations (Wasserman and Faust 1994, Chapter 12). Those countries who are very similar in their patterns are considered ‘equivalent’ and placed in the same positional block. Determining which countries are equivalent involves the use of an equivalence criterion; whereas earlier studies made use of a strict equivalence criterion known as ‘structural equivalence’ (e.g. Nemeth and Smith 1985; Snyder and Kick 1979), later studies used a more general criterion called regular equivalence (Mahutga 2006; Mahutga and Smith 2011; Smith and White 1992; Van Rossem 1996 xx Prell 2014 xx). Regular equivalence identifies countries who have similar patterns of ties to similar (rather than identical) others. For example, the U.S. and the U.K. may not import electronics from the exact same countries (although they might), but if they import electronics from countries who share similar trade characteristics (e.g. from countries who export electronics), then the U.S. and U.K. could be considered, in this instance, regularly equivalent. The more often this similarity occurs across the different trade relations, the more similar and hence ‘equivalent’ are the two countries.

In using regular equivalence as our criterion, we took the following steps in UCINET (Borgatti, et al. 2002). First, we made use of the REGE algorithm found in UCINET to ascertain the regular equivalences between all pairs of countries across the (normalized) six networks (see Wasserman and Faust 1994, Chapter 12 and Ziberna 2008 for technical details). This procedure resulted in a single, country-by-country equivalence matrix, where cell values ranged between 0 and 1: a score of 0 would indicate no equivalence between pairs and a score of 1 would indicate perfect equivalence.

This equivalence matrix was then submitted to a complete link hierarchical clustering routine (see Borgatti 1994 and Johnson 1967 for technical details). The procedure clusters similar countries together based on their equivalence scores, and displays these results as a tree diagram to assist in making heuristic decisions regarding the assignment of countries to positional blocks. In addition, we submitted the equivalence matrix to a multidimensional scaling (MDS) procedure. This procedure replicates the approach discussed by Gower (1966); essentially, it is a means to visualize the equivalences of pairs of actors in a Euclidean space. As such, equivalence scores for pairs of countries are translated into a set of estimated Euclidean distances, which are expressed as coordinates. These coordinates can be inputted into a visualization package (we used NetDraw in UCINET) to show countries in a two-dimensional space. Countries that are more equivalent are shown closer together in space, with further distances between country-points indicate decreasing levels of equivalence (see also Wasserman and Faust 1994, 287-88 for further details on MDS and other applications of MDS to network data).

We then superimposed our categorical network positions gained from our cluster analysis onto the MDS visualization of countries’ equivalence scores. Doing so acted as a cross-check and helped us finalize countries’ positions into 4 categories of core (4), semi-core (3), semi-periphery (2), and periphery (1) blocks.

**Table A. Pollution Outcomes Regressed on GDP Per Capita.**

|  | Production-based SO2 (ln) | | Consumption-based SO2 (ln) | |
| --- | --- | --- | --- | --- |
|  | Model 1 | Model 2 | Model 3 | Model 4 |
|  | *b* | *b* | *b* | *b* |
| GDP/capita (ln) | 0.348*** | 0.018 | 0.348*** | 0.178* |
|  | (0.0312) | (0.100) | (0.024) | (0.099) |
| Pop (1000s) (ln) |  | 0.521*** |  | 0.577*** |
|  |  | (0.092) |  | (0.158) |
| Urbanization (ln) |  | 0.001 |  | -0.001 |
|  |  | (0.003) |  | (0.004) |
| Constant | 1.845*** | 1.574** | 1.928*** | 0.923 |
|  | (0.276) | (0.568) | (0.242) | (0.788) |
| Fixed? | None^+^ | Country^#^ | None^+^ | Country^#^ |
| Observations | 3483 | 3,483 | 3588 | 3,588 |
| Wald χ^2^  R-squared | 123.79  0.552 | 249150.05  0.964 | 206.77  0.574 | 25279.98  0.959 |

* p < 0.10, ** p < 0.05, *** p < 0.01. These are unstandardized *b* values. Standard errors in parentheses.

^+^ The introduction of either/both the country and/or year fixed effect(s) led to a highly singular variance matrix, implying high collinearity. Thus, they were not included for this model.

^#^ The introduction of year fixed effect led to a highly singular variance matrix, implying high collinearity. Thus, it not included for this model.

**Table B. Mortality Outcomes Regressed on GDP Per Capita**

|  | Under Age 5  Mortality per 1000 | | Infant  Mortality per 1000 | |
| --- | --- | --- | --- | --- |
|  | Model 1 | Model 2 | Model 3 | Model 4 |
|  | *b* | *b* | *b* | *b* |
| GDP/capita (ln) | -0.534*** | -0.356*** | -0.527*** | -0.382*** |
|  | (0.015) | (0.011) | (0.015) | (0.010) |
| SO2EV ratio |  | -0.020** |  | 0.003 |
|  |  | (0.007) |  | (0.005) |
| Production-based SO2 (ln) |  | 0.013*** |  | -0.002 |
|  |  | (0.004) |  | (0.003) |
| Fertility Rate (ln) |  | 0.880*** |  | 0.713*** |
|  |  | (0.039) |  | (0.035) |
| Health % GDP (ln) |  | -0.023*** |  | -0.027*** |
|  |  | (0.005) |  | (0.005) |
| Urbanization (ln) |  | -0.005*** |  | -0.001*** |
|  |  | (0.001) |  | (0.001) |
| constant |  | 5.675*** | 7.624*** | 5.728*** |
|  |  | (.1103) | (0.1151) | (0.101) |
| Fixed? | Year^#^ | Year^#^ | Year^#^ | Year^#^ |
| N or Observations | 3,609 | 2,567 | 3,609 | 2,567 |
| Wald χ^2^ | 1420161.77 | 65417.93 | 849194.86 | 56453.52 |
| Adjusted R^2^ | 0.922 | 0.936 | 0.938 | 0.957 |

* p < 0.10, ** p < 0.05, *** p < 0.01. These are unstandardized *b* values. Standard errors in parentheses.

^#^ The introduction of country fixed effect led to a highly singular variance matrix, implying high collinearity.

**Table C. Pollution Outcomes Regressed on Categorical Network Position**

|  | Production-based SO2 | | Consumption based | |
| --- | --- | --- | --- | --- |
|  | Model 1 | Model 2 | Model 3 | Model 4 |
|  | *b* | *b* |  | *b* |
| Categorical network position | 0.016* | 0.057*** | 0.495*** | 0.027** |
|  | (0.009) | (0.012) | (0.0833) | (0.011) |
| Pop (1000s) (ln) |  | 0.963*** |  | 0.606*** |
|  |  | (0.008) |  | (0.152) |
| Urbanization (ln) |  | 0.036*** |  | 0.004 |
|  |  | (0.001) |  | (0.004) |
| Constant | 3.324*** | 0.975*** | 3.812*** | 1.706*** |
|  | (0.037) | (0.063) | (0.150) | (0.531) |
| Fixed? | Year, Country | Year^#^ | Year^#^ | Country^+^ |
| Observations | 3,486 | 3,483 | 3,591 | 3,588 |
| Wald χ^2^  R-squared | 127355.72  0.962 | 115836.40  0.819 | 35.37  0.475 | 87543.42  0.962 |

* p < 0.10, ** p < 0.05, *** p < 0.01. These are unstandardized *b* values. Standard errors in parentheses.

^#^ The introduction of country fixed effect led to a highly singular variance matrix, implying high collinearity.

^+^ The introduction of year fixed effect led to a highly singular variance matrix, implying high collinearity.

**Table D. Mortality Outcomes Regressed on Categorical Network Position**

|  | Under Age 5  Mortality per 1000 | | Infant  Mortality per 1000 | |
| --- | --- | --- | --- | --- |
|  | Model 1 | Model 2 | Model 3 | Model 4 |
|  | *b* | *b* | *b* | *b* |
| Categorical network position | 0.000 | -0.0133** | -0.131*** | -0.0176*** |
|  | (0.001) | (0.005) | (.027) | (0.006) |
| SO2EV ratio (ln) |  | 0.0705*** |  | 0.102*** |
|  |  | (0.009) |  | (0.008) |
| Production-based SO2 (ln) |  | -0.005 |  | -0.019*** |
|  |  | (0.006) |  | (0.005) |
| Fertility Rate (ln) |  | 1.252*** |  | 1.132*** |
|  |  | (0.053) |  | (0.047) |
| Health % GDP (ln) |  | -0.034*** |  | -0.046*** |
|  |  | (0.007) |  | (0.007) |
| Urbanization (ln) |  | -0.0161*** |  | -0.014*** |
|  |  | (0.001) |  | (0.001) |
| constant | 5.29*** | 3.183*** | 3.716*** | 3.014*** |
|  | (0.016) | (0.081) | (0.049) | (0.068) |
| Fixed? | Year, country | Year^#^ | Year^#^ | Year^#^ |
| N or Observations | 3,612 | 2,567 | 3,612 | 2,567 |
| Wald χ^2^ | 1169569.91 | 19884.18 | 23.55 | 21472.68 |
| Adjusted R^2^ | 0.989 | 0.924 | 0.705 | 0.934 |

* p < 0.10, ** p < 0.05, *** p < 0.01. These are unstandardized *b* values. Standard errors in parentheses.

^#^ The introduction of country fixed effect led to a highly singular variance matrix, implying high collinearity.
